# Supplementary material for: Recombinant Human Annexin A5 Ameliorates Localized Scleroderma by Inhibiting the Activation of Fibroblasts and Macrophages
Source: Pharmaceutics. 2025 Jul 30;17(8):986. doi: 10.3390/pharmaceutics17080986 (PMC12389603; doi:10.3390/pharmaceutics17080986)
Supplement: Supplementary file 1 [file pharmaceutics-17-00986-s001.zip › Supplementary figures.pdf]

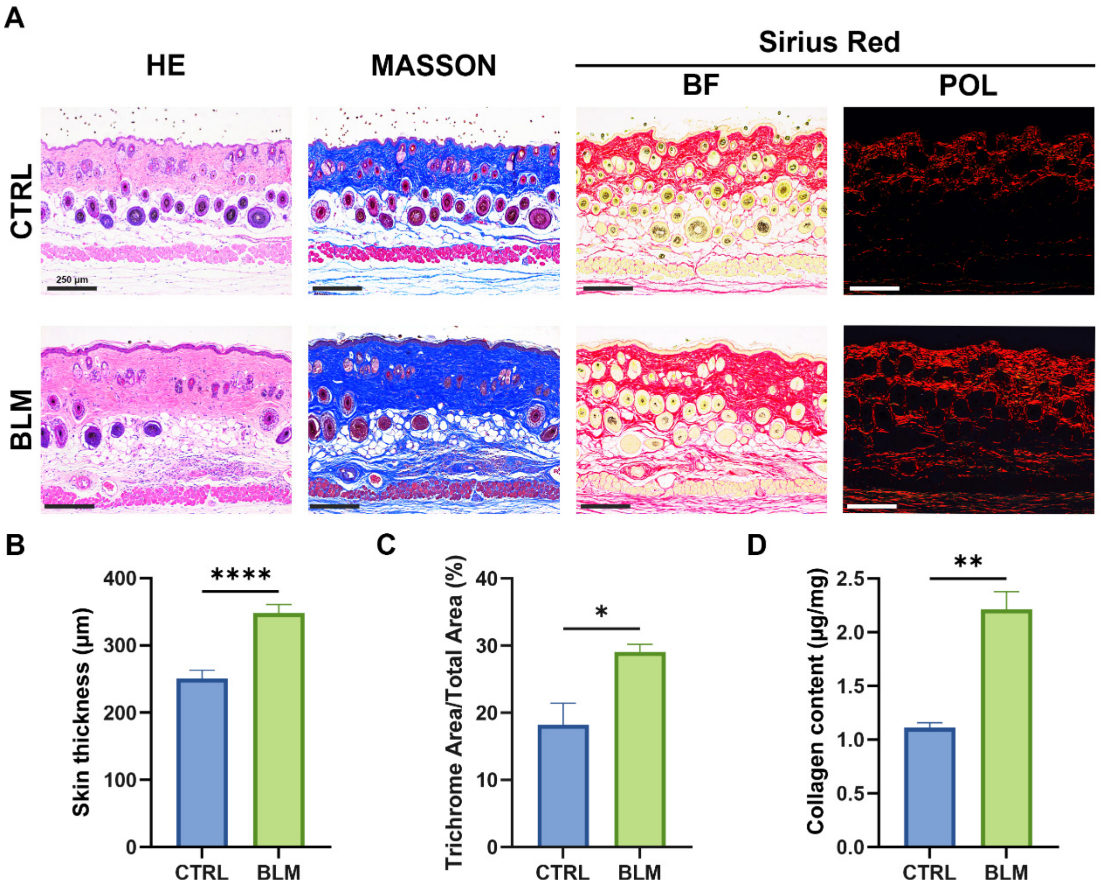

Supplementary Figure S1: The effect of bleomycin on skin fibrosis after 3 weeks. A. Representative images of HE staining, Masson's trichrome staining and Sirius Red staining (under bright field and polarized light device) for the skin tissue from the CTRL group and BLM group at 6 weeks. B. Quantitative analysis of full skin thickness (μm) of the skin tissue. C. Quantitative analysis of percentage of collagen-stained area of the skin tissue through Masson's trichrome staining. D. Quantitative analysis of collagen content (μg/mg) of the skin tissue by detecting the hydroxyproline content. Data are presented as mean ± SEM, with n=3 for each group. Statistical analyses were performed by comparing to the BLM group, where *p<0.05, **p<0.01, ***p<0.001, ****p<0.0001.

Figure 1. D

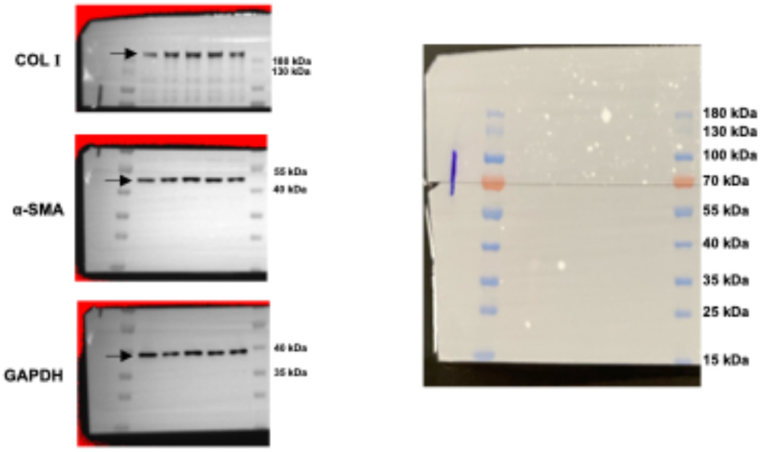

Figure 2. A

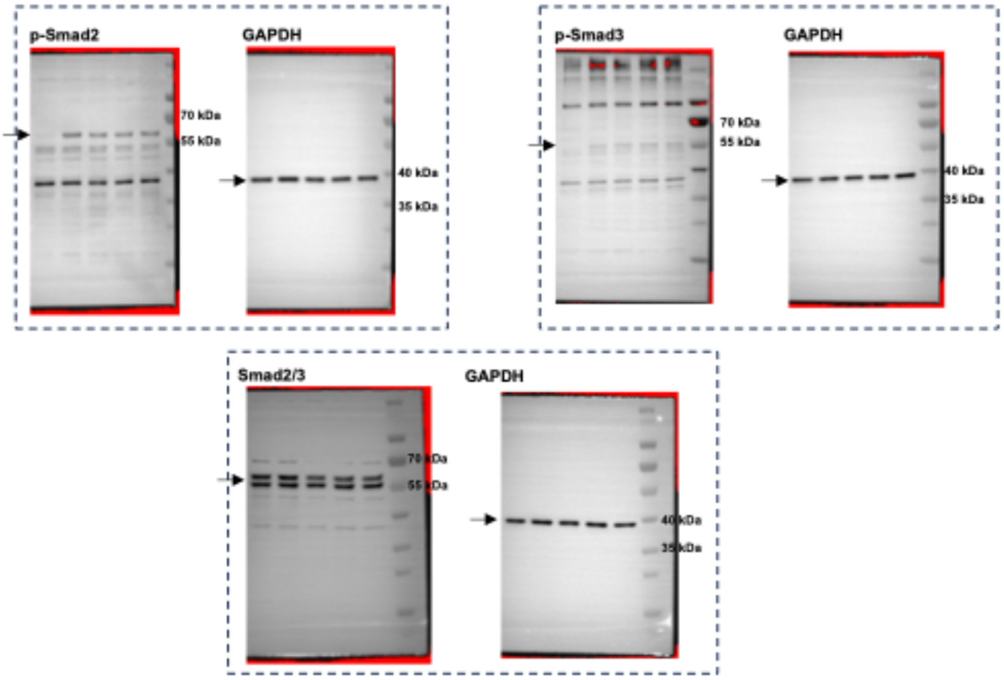

Supplementary Figure S2: Uncropped western blotting images.
