# Supplementary material for: Recombinant Human Annexin A5 Ameliorates Localized Scleroderma by Inhibiting the Activation of Fibroblasts and Macrophages
Source: Pharmaceutics. 2025 Jul 30;17(8):986. doi: 10.3390/pharmaceutics17080986 (PMC12389603; doi:10.3390/pharmaceutics17080986)
Supplement: Supplementary file 1 [file pharmaceutics-17-00986-s001.zip › Supplementary table.pdf]

Supplementary Table S1. Primer sequences for qRT-PCR.

Gene	Sequence	
Human COL1A1	Forward	AGTGTGGCCCAGAAGAACTG
	Reverse	GCCATACTCGAACTGGAATCCA
Human FN1	Forward	ATTCATGGGAGAAGTATGTGCATG
	Reverse	AGGACCACTTGAGCTTGGATAG
Human MMP1	Forward	CTACCCGGAAGTTGAGCTCA
	Reverse	AGCCCAGTACTTATTCCCTTTGA
Human TIMP-1	Forward	GCAGGATGGACTCTTGCACA
	Reverse	AACAGGGAAACACTGTGCATTC
Human TGFB1	Forward	AACCGGCCTTTCCTGCTTC
	Reverse	CAGTTCTTCTCCGTGGAGCTG
Human ACTA2	Forward	TCCTCCCTTGAGAAGAGTTACGA
	Reverse	CAGCAGACTCCATCCCGATG
Human CTGF	Forward	ATGGACGTTCGTCTGCCCA
	Reverse	TGTCTTCCAGTCGGTAAGCCG
Human GAPDH	Forward	TGGTATCGTGGAAGGACTCATGAC
	Reverse	ATGCCAGTGAGCTTCCCGTTCAGC
